# Infective Endocarditis Presenting as Unexplained Iron Deficiency Anaemia: A Diagnostic Challenge

**DOI:** 10.7759/cureus.89438

**Published:** 2025-08-05

**Authors:** Muhammad Arslan Ghous, Ahmed Malik Abuelgasim Malik, Yaman Dhary, Zahra R Almansoor, Krishna Girotra

**Affiliations:** 1 Acute Internal Medicine, University Hospitals of North Midland, Royal Stoke University Hospital, Stoke-on-Trent, GBR

**Keywords:** cardiac surgery, diagnostic challenge, enterococcus faecalis, infective endocarditis, iron deficiency anemia, mitral valve regurgitation, transoesophageal echocardiography

## Abstract

Infective endocarditis is a potentially fatal condition that can present with non-specific symptoms and rare hematologic manifestations, posing significant diagnostic challenges. We report a compelling case of a 67-year-old male with a history of type 2 diabetes, hypertension, and hyperlipidemia who sought medical attention for a five-month history of progressive iron deficiency anemia, accompanied by weight loss, fatigue, and vague constitutional symptoms. Initial extensive workup, including computed tomography of the thorax, abdomen, and pelvis, gastroscopy, colonoscopy, and transthoracic echocardiography, failed to identify an underlying cause. Despite these unrevealing results, persistent clinical suspicion for an occult process led to advanced cardiac imaging. Transoesophageal echocardiography and computed tomography coronary angiogram ultimately uncovered infective endocarditis, characterized by severe mitral valve regurgitation and trivial aortic regurgitation. Blood cultures concurrently confirmed Enterococcus faecalis bacteremia. The patient underwent urgent surgical intervention with mitral valve replacement and aortic valve repair, resulting in full resolution of his anemia and systemic symptoms. This case underscores the importance of maintaining a high index of suspicion for infective endocarditis in patients with unexplained iron deficiency anemia and non-specific complaints, even when preliminary cardiac imaging is normal. It highlights the pivotal role of advanced imaging modalities in securing a diagnosis and enabling timely, life-saving treatment.

## Introduction

Infective endocarditis (IE) is a life-threatening infection of the endocardium and heart valves, frequently caused by bacterial pathogens such as Staphylococcus aureus, Streptococcus species, and Enterococcus faecalis [1]. The clinical presentation of IE is often insidious, with nonspecific symptoms like fever, malaise, and weight loss [2]. Prompt diagnosis is crucial, yet can be elusive, especially when classical signs are absent or when initial imaging fails to reveal vegetations [3]. Iron deficiency anemia (IDA), defined by microcytic hypochromic anemia and low iron parameters, is commonly linked to gastrointestinal blood loss, dietary insufficiency, or malabsorption [4]. Chronic inflammation in IE may contribute to anemia of chronic disease and iron sequestration [5]. Rarely, IE may present with IDA, particularly when vegetations cause minor blood loss or systemic inflammatory responses. We present a rare case in which persistent IDA and vague systemic symptoms were the initial and only clues to an underlying diagnosis of IE, highlighting the need for comprehensive evaluation and the critical role of advanced imaging.

## Case presentation

A 67-year-old male with a history of type 2 diabetes mellitus, hypercholesterolemia, hypertension, and previous basal cell carcinoma presented in early 2023 with new-onset microcytic anemia. His hemoglobin declined from >130 g/L to 75-80 g/L over five months, with mean corpuscular volume (MCV) decreasing to ~80 fL. Iron studies showed low serum iron (3 µmol/L), low transferrin (1.76 g/L), low transferrin saturation (11%), and normal ferritin (347 µg/L) (Table 1). He reported weight loss (~6.35 kg), anorexia, and fatigue. Fecal immunochemical test (FIT) was negative.

**Table 1:** Summary of Blood Test Evaluation including hematology (FBC and differential), biochemistry (iron studies, vitamins, CRP, LFT, VBG, U&E), coagulation profile, and tumor markers. Results are presented with patient values, units, and reference ranges. Abnormal values are marked (↓ below, ↑ above normal). Estimated GFR is included without a reference range, as it is age- and creatinine-dependent. These findings aided assessment of inflammation, nutritional status, organ function, and malignancy risk. GFR: glomerular filtration rate, INR: international normalised ratio, APPT: activated partial thromboplastin time

Category	Component	Value	Range
Full Blood Count (FBC) and Differential	Haemoglobin	89 g/L	120 - 170 ↓ (L)
	White Cell Count	10.5 x 10⁹/L	4.0 - 11.0
	Platelet count	561 x 10⁹/L	150 - 450 ↑ (H)
	Red Cell Count	3.45 x 10¹²/L	4.30 - 5.70 ↓ (L)
	Haematocrit	0.273	0.37 - 0.50 ↓ (L)
	Mean Cell Volume	79.0 fL	80 - 100 ↓ (L)
	Mean Cell Haemoglobin	25.8 pg	26.5 - 31.5 ↓ (L)
	Neutrophil count	8.40 x 10⁹/L	2.0 - 7.5 ↑ (H)
	Lymphocyte count	1.20 x 10⁹/L	1.10 - 3.60
	Monocyte count	0.50 x 10⁹/L	0.20 - 0.80
	Eosinophil count	0.10 x 10⁹/L	0.04 - 0.40
Liver Function Test (LFT)	Alanine Transaminase	30 IU/L	0 - 48
	Aspartate Transaminase	27 IU/L	0 - 31
	Gamma Glutamyl Transferase	32 U/L	0 - 50
	Total Bilirubin	6 µmol/L	0 - 21
	Albumin	26 g/L	35 - 50 ↓ (L)
Urea and Electrolytes (U&E)	Sodium	136 mmol/L	133 - 146
	Potassium	5.4 mmol/L	3.5 - 5.3 ↑ (H)
	Urea	7.8 mmol/L	2.5 - 7.8
	Estimated GFR	67 mL/min/1.7	
	Creatinine	100 µ enmol/L	59 - 104
C-Reactive Protein (CRP)	CRP	84.1 mg/L	0 - 5 ↑ (H)
Tumor Markers	Prostate Specific Antigen	0.84 ng/mL	0 - 4.5
Coagulation Screen	Prothrombin INR	1.1	0.80 - 1.20
	APTT Ratio	1.01	0.80 - 1.17
Iron Studies	Serum Iron	3 µmol/L	13 - 32 ↓ (L)
	Ferritin	347 ng/mL	20 - 250 ↑ (H)
	Transferrin	1.76 g/L	2.0 - 3.6 ↓ (L)
	Thyroid Stimulating Hormone	1.31 mU/L	0.1 - 5.0
Vitamins & Folate	Vitamin B12	324 pmol/L	180 - 900
	Serum Folate	6.2 nmol/L	7.0 - 45.0 ↓ (L)
	Vitamin D	81 nmol/L	75 - 250
Venous Blood Gas (VBG)	pH	7.38	7.35 - 7.45
	pCO2	4.96 kPa	4.8 - 6.0
	pO2	4.78 kPa	10.0 - 13.5 ↓ (L)
	Lactate	1.3 mmol/L	0.6 - 2.5
	Glucose	4.9 mmol/L	3.5 - 9.0
	Standard Bicarbonate	21.88 mmol/L	22 - 26 ↓ (L)

**Figure 1:**
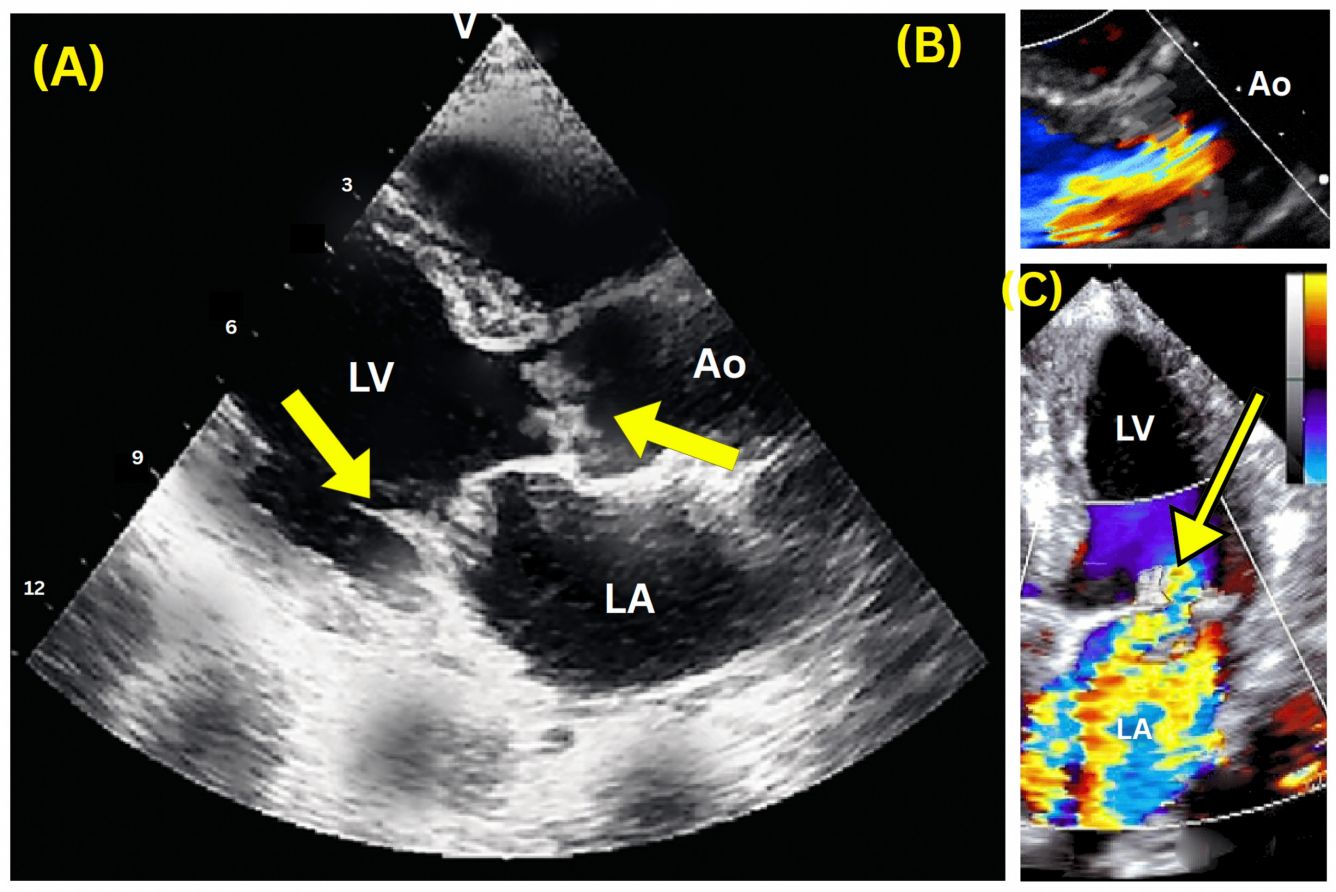
Transoesophageal Echocardiography (A) Two-dimensional transoesophageal echocardiography (TOE) image showing vegetations on the mitral and aortic valves, with the left ventricle (LV), left atrium (LA), and aorta (Ao) labeled. Yellow arrows indicate the presence of vegetations on the mitral valve and the aortic valve. (B) Colour Doppler imaging of the aortic valve revealing trivial aortic regurgitation, with a small jet of retrograde flow (yellow and blue turbulence) from the Ao into the LV. (C) Colour Doppler imaging of the mitral valve demonstrating severe mitral regurgitation, with a significant jet of retrograde flow (yellow and blue turbulence) from the LV into the LA.

Extensive malignancy workup included CT thorax, abdomen, and pelvis; gastroscopy; and colonoscopy. These showed pan-colonic diverticulosis and benign caecal polyps, with no malignancy. Coeliac disease was excluded. Initial transthoracic echocardiogram (TTE) was unremarkable. In September 2023, he was referred to Ambulatory Emergency Care due to elevated inflammatory markers and systemic symptoms (fevers, night sweats, dyspnea). Examination revealed a pansystolic murmur. ECG showed right bundle branch block. Empirical IV amoxicillin (2 g four times a day) was commenced after obtaining blood cultures. TTE showed no vegetations or systolic dysfunction. However, transoesophageal echocardiography (TOE) revealed severe mitral regurgitation, vegetations on the anterior mitral leaflet and non-coronary cusp of the aortic valve, and trivial aortic regurgitation (Figure 1). Blood cultures grew *Enterococcus faecalis*. CT coronary angiogram confirmed vegetations and a probable splenic infarct. Repeat CT revealed splenic and renal infarcts and bilateral pleural effusions.

An endocarditis multidisciplinary team recommended urgent mitral valve replacement, aortic valve repair, and possible coronary artery bypass graft surgery (CABG). The patient underwent mitral valve replacement with a 27 mm St Jude’s mechanical valve, aortic valve repair, and pleural effusion drainage. Postoperatively, he completed four weeks of IV amoxicillin and ceftriaxone, followed by four weeks of oral amoxicillin.

Hemoglobin stabilized to 120 g/L postoperatively. Iron indices improved (serum iron 9 µmol/L, transferrin 2.03 g/L, ferritin 244 µg/L). No recurrent symptoms were reported. The patient returned to baseline functional status and remained stable at follow-up.

## Discussion

IE can mimic numerous other conditions, and its presentation as isolated IDA is rare. In this patient, the absence of classical signs and an unremarkable TTE delayed the diagnosis. The elevated ferritin in the setting of IDA reflects an inflammatory component, consistent with anemia of chronic disease complicating true IDA [6]. This case emphasizes the importance of thorough cardiac evaluation, especially in patients with unexplained anemia and systemic symptoms. TOE has superior sensitivity to TTE in detecting vegetations, particularly in patients with prosthetic valves or suboptimal TTE windows [7]. The detection of *E. faecalis* bacteremia, a known cause of IE, further guided the diagnostic process [8]. Early recognition and surgical intervention are crucial in IE, particularly with heart failure, uncontrolled infection, or embolic phenomena. Our patient's favorable outcome reflects timely multidisciplinary collaboration and the utility of advanced imaging and prompt surgery.

## Conclusions

Unexplained iron deficiency anemia, especially when persistent and accompanied by systemic symptoms, should prompt consideration of infective endocarditis, even in the absence of initial echocardiographic evidence. Advanced imaging modalities such as TOE and CT coronary angiography can be pivotal in diagnosis. This case underscores the importance of clinical vigilance and multidisciplinary management in achieving successful outcomes in complex presentations of IE.
